# Traumatic brain injury and rTMS-ERPs: Case report and literature review

**DOI:** 10.1515/biol-2022-0677

**Published:** 2023-09-13

**Authors:** Yutong Fu, Chunyan Xu, Hong Fan, Xue Yang, Jibing Ou, Liqing Yao, Wenli Wang

**Affiliations:** Department of Rehabilitation Medicine, The Second Affiliated Hospital of Kunming Medical University, Kunming, Yunnan Province, China

**Keywords:** transcranial magnetic stimulation, event-related potentials, rehabilitation, cognition, latency, amplitude, P300

## Abstract

Currently, there are no cases of targeted, individualized repeated transcranial magnetic stimulation (rTMS) treatment based on event-related potential (ERPs) results showing the activation of functional brain regions. The identification and treatment of mild cognitive impairment after traumatic brain injury are challenging. rTMS has shown unique advantages in previous studies, with positive effects on noninvasive modulation and neuroplasticity after brain injury. The selection of the rTMS parameters and targets remains controversial. ERPs indicate the cortical activity involved in cognitive processing in patients. Therefore, this study proposes that ERPs can be used as biomarkers of cognitive recovery. The results of this study will guide the development of rTMS protocols for patient treatment. To help clinicians better apply rTMS and ERPs in combination, we conducted a relevant literature review and discussion, detailing the therapeutic mechanisms of the combination of ERPs and rTMS. This will facilitate the precise assessment and personalized treatment of such patients, improve the abnormal processing patterns of patients, and promote their return to life and society.

## Graphical abstract

**Figure:**
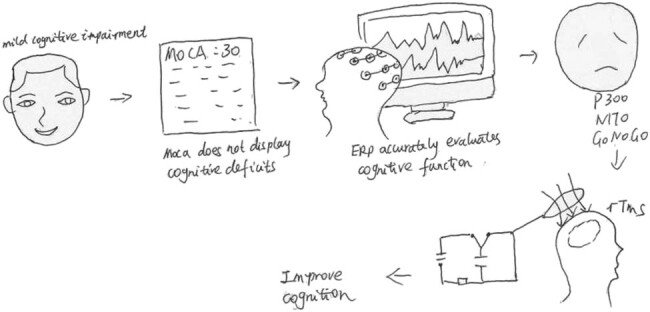

## 1 Background

Traumatic brain injuries (TBI) can result from blows or bumps to the head. Most people with brain injury are young and of prime age, with 300–400 cases per 100,000 people between the ages of approximately 15 and 24 [1,2]. The effects of TBI include impairments in cognition, behavior, and emotional functioning, which can cause care and financial burdens for families and society. Depending on the severity of TBI, approximately 49% of patients with moderate-to-severe TBI, 34% with mild TBI [3], and mild cognitive impairment due to injury are not usually detected. Occupational therapy is used to improve cognitive function but usually has a limited effect. Repeated transcranial magnetic stimulation (rTMS) is a noninvasive neurophysiological technique that transmits a series of pulses in a rhythmic repetitive form that regulates neural activity and cortical excitability. Preliminary studies have shown that rTMS is safe, tolerable, and effective. rTMS modulates cortical excitability depending on stimulus parameters. Low-frequency (1.0 Hz) rTMS reduces cortical excitability, whereas high-frequency (>1.0 Hz) rTMS increases cortical excitability.

Because the initial event-related potential (ERP) results in this patient showed frontal hyperactivation, reduced parieto-occipital connectivity, cognitive decline, and significant depression; low-frequency rTMS stimulation of the right dorsolateral prefrontal cortex (DLPFC) was expected to correct this imbalance, treat cognitive impairment, and improve depression. There are few studies on low-frequency rTMS stimulation of the right DLPFC, mostly in small samples [4], and its efficacy is not yet clear. Decreased emotional regulation is observed in patients with mild cognitive impairment [5]. Patients with mild cognitive impairment and depression have abnormally high activity in the right prefrontal cortex, and low-frequency transcranial magnetic stimulation of the right DLPFC for depression may correct the imbalance between the right and left prefrontal activity, leading to normalization [6,7]. Most previous studies have used high-frequency left DLPFC, whereas low-frequency right stimulation is better tolerated and may reduce the risk of seizures compared with high-frequency left stimulation. Therefore, a treatment protocol for the right DLPFC at 1 Hz was proposed for use in this study. The therapeutic effect of rTMS is region-specific: rTMS stimulation of the prefrontal lobe improves cognitive function and is a neuromodulatory method that produces long-term effects. rTMS pulses induce currents within the cortex beneath the stimulation site [8], leading to local neural activation [9] as well as changes within distributed networks. Subsequent effects may exceed the duration of stimulation, and post-TMS effects should be temporally and spatially dynamic within local areas and whole-brain networks [10]. Stimulation of the right DLPFC also indirectly stimulates the ventrolateral prefrontal cortex (VLPFC), which affects the limbic system, thereby improving executive and inhibitory functions and depressed mood.

Common imaging examinations, including computed tomography (CT) and magnetic resonance imaging (MRI), can only identify structural damage in the brain and cannot detect cognitive impairments after TBI. Approximately 67% of patients with neurocognitive disorders post-TBI did not show significant changes in brain structure at the time of evaluation but showed abnormal behavior and cognitive impairment. There is a clear need for more sensitive brain imaging techniques to detect the structure and function of a patient’s brain [11]. ERPs can dynamically observe the cognitive processing of patients in a time-dependent manner, providing a basis for individualized and precise treatment. Compared with behavioral measurements, the main advantages of ERPs are (1) higher temporal resolution and (2) better sensitivity and specificity. Therefore, we treated a young patient with TBI for 1 year and found that the sensitivity of ERPs neural markers for assessing cognitive function was better than the Montreal Cognitive Assessment (MOCA) scale, and rTMS neuromodulation treatment was performed according to the ERP results to identify the effectiveness of rTMS treatment using right DLPFC at 1 Hz, which will hopefully be used in the future in such patients.

## 2 Case presentation

**Figure 1:**
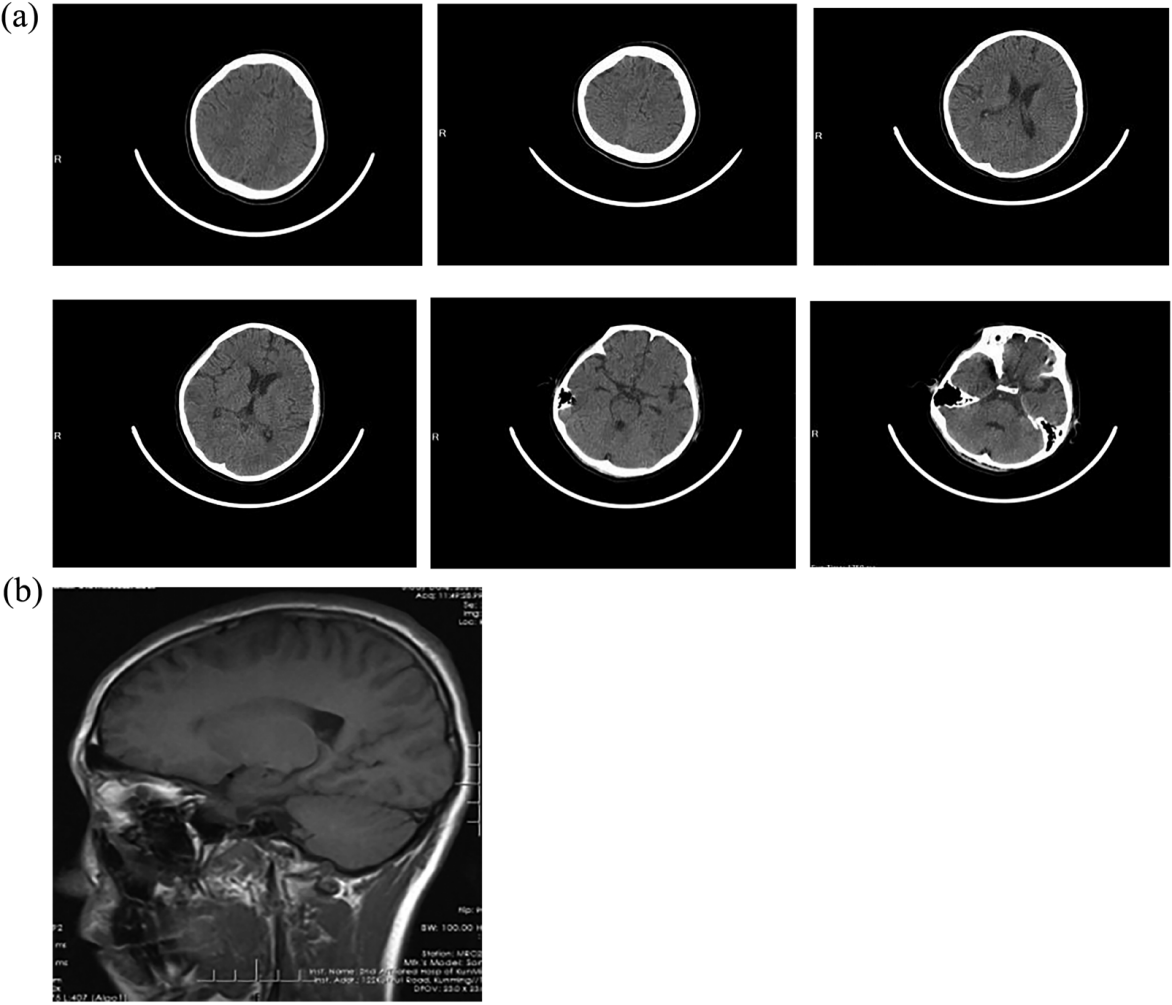
(a) CT image and (b) MRI image.

The TBI inclusion criteria included a history of direct head trauma or indirect trauma with post-injury symptoms such as coma and vomiting. The diagnosis was confirmed by CT and MRI. The exclusion criteria were serious central system infection; serious heart, lung, liver, kidney, and other important organ diseases; serious multiple injuries; surgery due to changes in condition during treatment; and coma caused by primary brainstem injury. The patient was male, 18 years old, with high school education or above, Tibetan, right-handed. He fell from a height on 21 November 2020, and was immediately comatose. The detailed injury process could not be recalled. Based on the CT and MRI findings of the head, a diffuse axonal injury was suspected. He underwent tracheostomy on 30 November 2020, due to a pulmonary infection. The patient was then transferred to a hospital, and internal fixation was performed for pelvic fracture on 17 December 2020. The patient was transferred to the ICU postoperatively. The tracheal intubation was removed on 29 December 2020. In February 2021, the patient was admitted to the rehabilitation department and complained of a slow response combined with memory impairment and cognitive decline. He wished to return to campus, take care of himself, and attend an ideal university (Figure 1).

### 2.1 Treatment

He received 20 min of rTMS treatment every day, five times a week for 3 weeks; the treatment site was the dorsolateral area of the right prefrontal lobe (R-DLPFC), the frequency was 1 Hz, the threshold was 80%, the intensity was 16%, the number of stimuli was 5, the stimulation time was 5 s, the interval time was 1 s, the number of repetitions was 200, and the total number of stimuli was 1,000.

The patient underwent regular rehabilitation, including occupational and physical therapies. Treatment included physical and cognitive function training, and psychological guidance. Each program lasted for 40 min per day, five times per week.


**Informed consent:** Informed consent has been obtained from all individuals included in this study.
**Ethical approval:** The research related to human use has been complied with all the relevant national regulations, institutional policies and in accordance with the tenets of the Helsinki Declaration, and has been approved by the authors’ institutional review board or equivalent committee.

### 2.2 ERPs examination and analysis

Recordings were made on a 32-channel EEG, at 9 o’clock in the morning. The paradigms included the P300, N170, and GO/NOGO. The experimental material was edited using E-prime3.0, and the acquired data were analyzed offline using Curry8 software.

### 2.3 Clinical schedule

The patient was admitted to our hospital for rehabilitation for the first time in February 2021, underwent the first baseline assessment at admission, performed routine rehabilitation training combined with rTMS treatment for 3 weeks, underwent the first post-treatment evaluation, and again underwent rehabilitation training combined with rTMS treatment, followed by the second post-treatment evaluation. The patient was discharged. The patient was re-examined after 2 months and 6 months.

**Figure 2:**
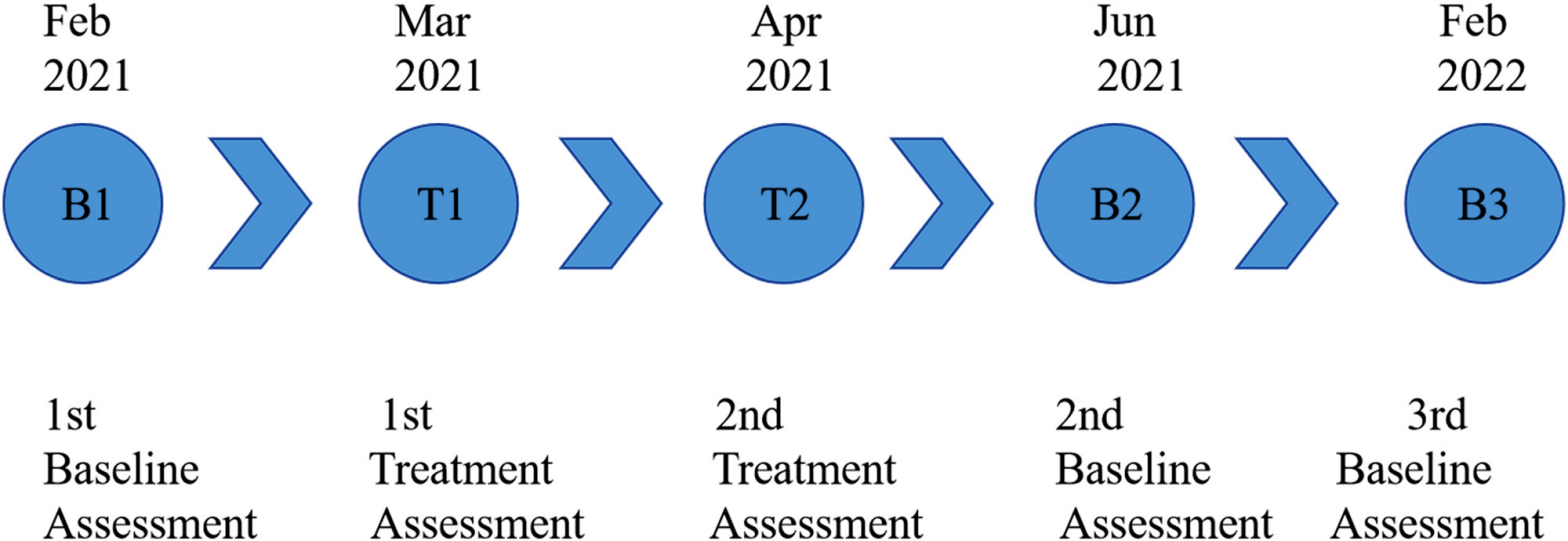
Overview of the experimental design and clinical schedule for the overall and current studies (B: baseline and T: treatment).

Figure 2 provides an overview of the clinical timeline.

### 2.4 Evaluation

The scales included the MoCA, Functional Independence Measure (FIM), Hamilton Depression Scale (HAMD), and Hamilton Anxiety Scale (HAMA) to assess the patients’ cognitive function, activities of daily living, depression, and anxiety.

**Figure 3:**
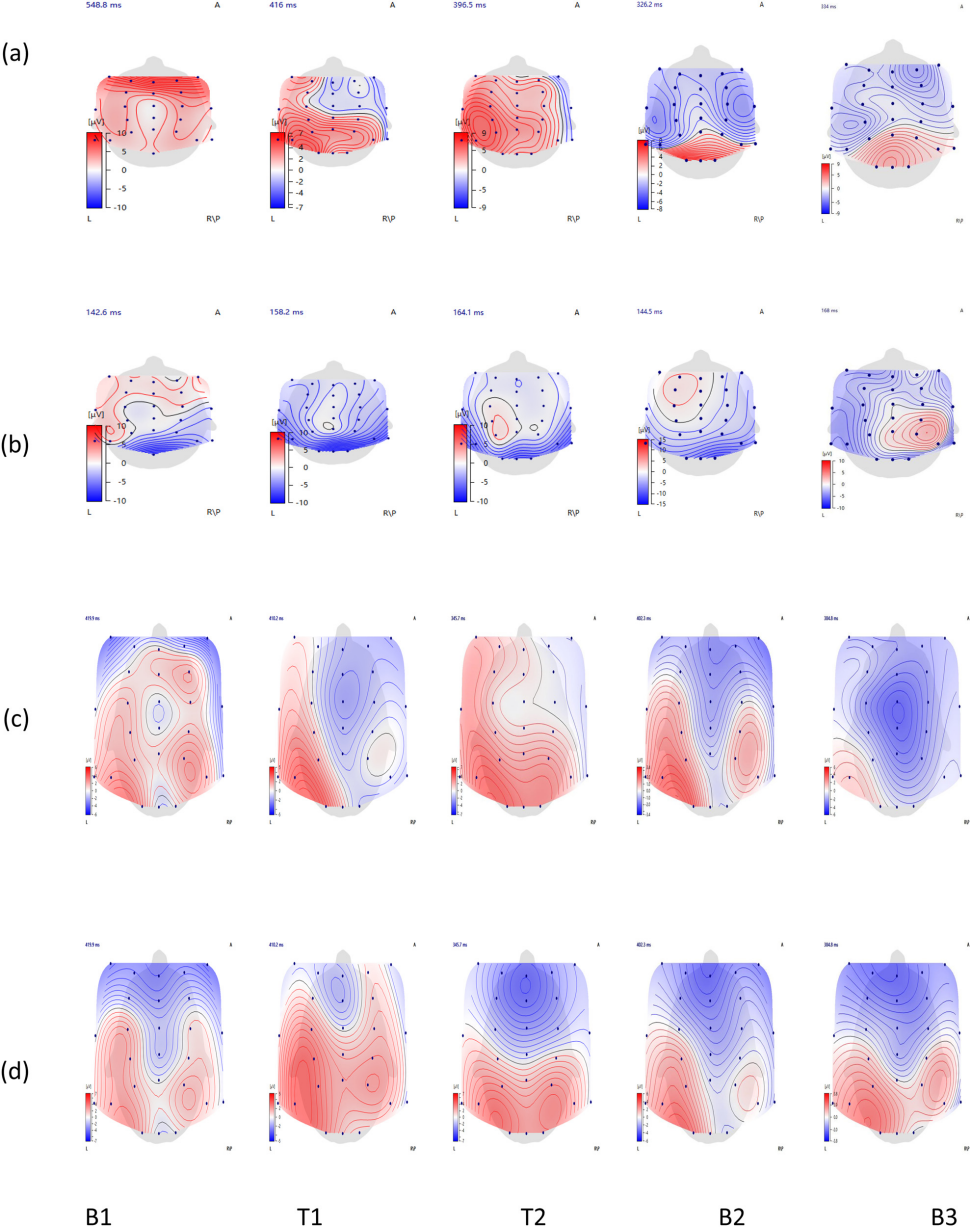
(a) Brain topographic map of P3-P300, (b) brain map of P8 position of inverted faces in N170, (c) brain topographic map of P3-GO, and (d) brain topographic map of P3-NOGO.

The assessment results are shown in Table 1. The ERP results are shown in Table 2. The topographic map of each component changed over time, as shown in Figure 3a–d.

**Table 1:** Scales results

Number	MOCA	FIM		HAMA	HAMD
	Score	Motor	Recognition	Score	Score
B1	28	52	29	7	24
T1	30	53	35	4	20
T2	30	79	35	2	15
B2	30	82	35	1	10
B3	30	82	35	1	3

**Table 2:** ERPs results

Number	P300			GONOGO P3-Go		GONOGO P3-Nogo			Inverted faces		Upright faces		Inverted house		Upright house		
	Latency	Amplitude	location	Latency	Amplitude	Latency	Amplitude	Location	Latency	Amplitude	Latency	Amplitude	Latency	Amplitude	Latency	Amplitude	location
B1	521.5	20.1	FZ	419.9	1.1	433.6	0.4	PZ	144.5	4.2	160.2	1.1	115.2	8.7	103.5	6.6	P8
T1	416.0	4.4	PZ	408.2	5.1	408.2	5.3	P7	158.2	8.7	156.3	8.7	123.0	5.5	127	5.0	P8
T2	396.5	6.6	PZ	345.7	2.8	345.7	1.9	PZ	164.1	6.9	152.3	3.6	156.3	4.0	123	0.1	P8
B2	326.2	0.2	PZ	402.3	3.3	402.3	4.3	P7	146.5	7.6	146.5	4.2	119.1	5.4	119.1	5.2	P8
B3	337.9	4.3	PZ	384.4	1.7	384.4	2.7	P7	136.7	0.5	152.3	5.8	119.1	3.3	123.0	10	P8

Latency: MS; Amplitude: UV.

## 3 Clinical results and outcomes

At time point B3, the MOCA was 30, with normal cognitive function, and FIM1 was 117, indicating full independence. The HAMA score was 1, the HAMD score was 3, and there were no anxiety or depressive symptoms. After two cycles of rTMS treatment, the patient’s cognitive function, motor function, depression, and activities of daily living significantly improved. The brain underwent plastic changes in the P3 component of P300, the brain topographic map showed that the frontal area is overactivated relative to parietal area activation, and the latency was shortened. The P3 amplitude was higher than that of NOGO, and the P3 amplitude of NOGO was larger than that of GO during the remainder of the monitoring period. The latency of the N170 component at T2 was longer than that at the other time points. Three months after the end of treatment, the activation of the P300 brain topography changed from extensive activation of the central and parietal areas to activation of the parietal and occipital areas. In the GO/NOGO brain topographic map, the P3-GO and NOGO, the activation area of the brain area was reduced, and the PZ activation was weakened. At 6-month follow-up, there was no change in the MOCA or FIM. In the N170, the amplitudes of inverted houses and faces decreased, with a corresponding decrease in the activation of P8, and the amplitudes of upright houses and faces increased, with a corresponding increase in P8 activation. The P3 amplitude of NOGO was still larger than that of GO; the activation area of the P3 component of GO was further reduced, limited to P7, and the latency did not change significantly compared with B2. Compared with the B2 time point, the latency of P3-P300 did not change significantly, and the activation patterns of the brain regions were also similar.

## 4 Discussion

### 4.1 Main findings

Patients with TBI will have hidden cognitive dysfunction; brain injury leads to an abnormal cognitive process; and ERPs can be used as cognitive biomarkers, supplementing the shortcomings of insufficient sensitivity to clinical scales, because even patients with a full MOCA score will show abnormal cognitive processing. According to the ERP results, targeted rTMS treatment was carried out on the patient in this case, which can effectively improve the inhibition ability, reaction speed, and plasticity changes in the brains of patients with brain trauma. Changes in perceptual processing in patients with TBI are unclear. Three to six months of follow-up revealed a decrease in inhibitory capacity and overall cognitive function, and a brain topographic map showed a narrower range of activation in the P300 and GO/NOGO brain regions.

### 4.2 ERPs and cognition

Patients with mild cognitive impairment often have a full MOCA score, which does not mean that the patient has no cognitive impairment, but rather that through repeated measurements the patient has memorized the answer. Existing cognitive assessment scales, such as the MOCA, cannot objectively and quantitatively display a patient’s cognitive processing and cannot sensitively detect patients with mild cognitive impairment. If such patients do not receive timely intervention, they have an increased risk of developing dementia in the future.

TBI involves complex and heterogeneous pathological changes that often result in long-term cognitive and behavioral impairments and disabilities. Diffuse axonal injury is one of the most common neuropathological consequences of TBI. Trauma causes damage to normal brain cells. The axis breaks through the bundles of white matter fibers in the affected brain, leading to diffuse axonal damage, which can lead to local swelling that slows down the transmission of signals. Cognitive impairment is a common outcome of TBI. The affected areas include information processing speed, attention, memory, and executive function.

The P300 can effectively reflect a patient’s cognitive processes, such as understanding and judgment based on attention, and its results are not affected by patient culture, language, or literacy. The P300 amplitude shows the resource mobilization of brain tissue in processing information, and the latency shows the speed at which the patient’s brain tissue recognizes, processes, and encodes external stimuli; the change in P300 occurs earlier than the clinical change [12]. Prolonged latency of P300 is a sensitive indicator of cognitive dysfunction. P300 in patients with TBI usually presents with an extended latency and decreased amplitude [13], mainly in the P3b component, as the apical distribution of P3b is associated with working memory and task cognitive resource allocation and a decrease in P3b is associated with decreased task performance [14]. In this study, the first P300 results showed that the FZ region was overly complicated and attenuated, and increased activation in this brain region is generally thought to reflect an increase in the processing load. The high activation patterns observed in patients with TBI may reflect “compensatory” processes. TBI altered participants’ processes of cognitive function regulation after injury. When P300 is used in combination with a cognitive scale, its sensitivity for diagnosing cognitive dysfunction is as high as 96%, and its specificity is 80%, which provides an effective method for evaluating patients with preclinical cognitive impairment. Studies have shown that P300 latency in patients with mild cognitive impairment is significantly longer, and this prolongation is positively correlated with the degree of cognitive impairment. Moreover, the P300 latency was significantly shortened after the patient in this study was treated with rTMS, which demonstrated that cognitive function had improved.

The ability to suppress reactions is an important part of executive function, and refers to the ability to ignore or suppress irrelevant or distracting information, including sensational and dominant automatic behaviors unrelated to the task at hand. This is essential for adapting to rapid changes in the environment as well as social interactions, such as when traffic lights turn red, requiring a driver to stop and wait, and many other situations that require stopping dominant responses and responding to suddenly changing signals, especially stopping an action that has just been initiated. Executive function represents a fundamental ability for daily cognitive activity [15], and response inhibition, which is the basis of multiple advanced cognitive functions of the brain, causes significant changes in early cognitive dysfunction. Reaction inhibition disorders may trigger impulsive behavior [5]. The GO/NOGO task included Go and No-go trials. The Go trial represents the tendency to respond, whereas the No-go trial requires response inhibition. Both the Go and No-go trials produced P3 components between 250 and 600 ms after stimulation, and the NOGO-P3 components were generally associated with the later stages of the inhibition process, which mainly reflected the inhibition ability [16]; the decrease in the P3 amplitude suggested that there was a response inhibition dysfunction. P3 latency reflects cognitive efficiency and processing progress, which is closely related to the conflict inhibition of the motor system in the prefrontal cortex, which is distributed in the central and parietal lobes of the scalp, is an indicator of response inhibition [17] and is the main indicator observed in this study. At the BI time point, both latency periods of P300-P3 and P3-GO/NOGO were prolonged, and a decrease in the speed of information processing may represent a core aspect of cognitive function recovery, which indicates an overall decrease in the patient’s information processing speed, executive function, attention, and overall cognitive performance. During the course of rTMS treatment, both the GO-P3 and NOGO-P3 showed significantly shorter latency, indicating an increase in cognitive function and speed of information processing. At T1, the amplitude of the NOGO-P3 significantly increased, indicating a significant improvement in inhibition. Unfortunately, the effect was not maintained for long after the end of treatment, and a rebound trend was observed at 3 and 6 months of follow-up.

The N170 paradigm mainly reflects the perceptual integration of facial features; the N170 is the negative deflection potential of the ERPs signal, which appears approximately 170 ms after the start of stimulation and peaks in the occipital lobe, especially the P8 position. The P8 electrode is close to the hippocampus and the right fusiform gyrus, which are important in face recognition; the visual evoked potential N170 amplitude caused by the inverted face is greater than that of the upright face [18], and N170 is associated with cognitive decline. In particular, patients experience a deterioration of early sensory processing mechanisms [19]. The amplitude and brain topographic maps of this patient showed decreased bilateral parietal temporal lobe region involvement, reflecting impairment in facial coding processing [20,21]. However, throughout the treatment period, the N170 levels did not show a significant change. This may be because rTMS had no significant effect on perceptual processing.

### 4.3 rTMS and cognition

Specific brain regions have specialized functions, and each region participates in an extended brain network. We are interested in combining brain stimulation with neurophysiological imaging, which can be used to study the interactions between extended networks of brain regions using this new causal approach. Brain stimulation affects not only the targeted local regions but also the activity of remotely interconnected regions to reveal dynamic changes in interactions between brain regions.

Traditional treatments for cognitive impairment are relatively ineffective and may be related to the pathophysiological changes that occur after brain injury. Structural and functional impairments of neural networks and cognitive function rely heavily on the combined activity of neural networks throughout the brain. TBI can damage structural and functional connections. rTMS stimulation affects the cerebral cortex and causes persistent changes in cortical excitability. rTMS treatment improves structural and functional changes throughout the neural network, such as increasing the physical integrity of the white matter bundles, structural connectivity, brain capacity [22], communication efficiency across the cortical network, and functional connectivity [23]. The spatial propagation of the rTMS post-effect in the cortico-subcortical network alters the activity of the target cortical region and its connected subcortical networks [24]. The rTMS post-effect is observed not only around the stimulated area but also in the remote area connected to the target, and changes in the plasticity of brain regions other than the frontal lobe can also be observed from the topographic map changes in this case. It is a noninvasive and well-tolerated neuromodulation method. This is the theoretical basis for the use of rTMS in the treatment of cognitive impairment after TBI.

Neural connections for cognitive function are usually found between regulatory networks involving the prefrontal and subcortical systems responsible for control and inhibition processes, such as the DLPFC and VLPFC. The DLPFC has important implications in a variety of higher-order cognitive functions. A new link in the relationship between cognitive control and emotion regulation was established when the DLPFC was activated, resulting in an excitatory change in the strength of the connection from the DLPFC to the VLPFC, which is responsible for assessing emotional stimuli and is also involved in response selection and inhibition [25]. Similarly, the increase in P300 amplitude in GO/NOGO after stimulation indicated an improvement in conflict resolution. This finding is consistent with previous findings [26]. In humans and other animals, the executive function is disrupted after brain injury involving the frontal cortex. The anterior cingulate gyrus and DLPFC are two regions used for cognitive inhibitory treatment of TBI in adolescents. The ability of the prefrontal cortex to inhibit executive function responses plays a key role [27]. Neuroimaging studies have described the role of the DLPFC in effective inhibition, and a reduction in DLPFC activation is consistent with a decrease in response inhibition capacity [28]. Previous studies have shown that after high-frequency stimulation of the DLPFC, executive function improved [29]. The selection of rTMS parameters needs to be further studied in the future to expand the sample size. When examined at B1, the participant in this study showed relatively high prefrontal cortex activity, so the dorsal lateral region of the right prefrontal lobe was suppressed at a frequency of 1 Hz. The inhibitory effect on the DLPFC has been used in many studies to improve cognitive dysfunction in patients with neurological disorders, including performance, learning, memory, and attention [30]. The therapeutic effects of rTMS are region-specific, with rTMS stimulation of the prefrontal lobe improving cognitive function [31], and it is a neuromodulation method that produces long-term results. The results of an EEG study in healthy subjects showed that when 1 Hz-rTMS is used on the right DLPFC, the α coherence of the prefrontal cortex increases significantly [32] and is directly involved in internal mechanisms of information processing, attention, and consciousness [33]. These findings suggest that the significant increase in α coherence of 1 Hz-rTMS in the right DLPFC to the prefrontal cortex may be related to the mechanism by which TMS treats depression [4]. In depressed patients, intra-frontal hemispheric interdependence was significantly reduced, while interfrontal hemispheric consistency in all bands was significantly reduced during emotional face processing. The results of the present study are consistent with these findings in patients with N170 abnormalities [5]. Previous studies have also shown that the frontal and parietal regions play important roles in emotion processing. The more severe the depressive symptoms, the more significant the increase in frontal nodal efficiency and decrease in parieto-occipital nodal efficiency [34].

rTMS at 1 Hz has been shown to reliably suppress local hemodynamic activity [35]. Although the local effects of stimulation can be understood using rTMS, how local changes in neural activity affect neural networks remains unclear. Studies combining transcranial magnetic stimulation with functional neuroimaging techniques have shown that rTMS can also affect distal brain regions associated with the target area of stimulation [36]. For example, modulatory pulses at cerebellar and frontal sites have similar effects on the primary motor cortex [37]. rTMS induces cortical changes beyond the duration of the stimulation itself, and there is growing evidence from human and animal studies that transcranial magnetic stimulation modulates neuronal activity beyond the site of stimulation, affecting a distributed network of brain regions, and that the therapeutic and behavioral effects of transcranial magnetic stimulation are mediated by this distributed network effect [38]. The effects of rTMS can be improved by targeted stimulation based on individual MRI anatomy. In the present study, the stimulation of the right DLPFC affected other cortical and subcortical brain regions. The three important components between the frontal-limbic system circuit, anterior cingulate cortex (ACC), prefrontal cortex, and limbic system include bottom-up ACC to dlPFC and top-down vmPFC and VLPFC connected to ACC paths [39]. Emotional regulation is mediated by a specific neural circuit that includes the frontal-limbic region, and abnormalities in this region are associated with depression.

There is evidence that mood regulation processes selected for low-frequency right-sided stimulation of the DLPFC may have superior effects to high-frequency left-sided stimulation [40]. ERP can help determine the causal relationship between cortical activity and behavior by inhibiting the right prefrontal dorsolateral area and achieving a balance of inhibition between the two hemispheres. The DLPFC plays a key role in mood regulation, especially in cognitive and behavioral control, and DLPFC neuromodulation alters the activation and functional connectivity between the DLPFC, ventral medial prefrontal cortex, and amygdala.

Analysis of P3 in the apical region revealed a significant effect of rTMS stimulation. In numerous studies, the P300 has been associated with behavioral success in performing tasks involving attention and memory [41]. This study not only showed a significant change in the P3 amplitude in the frontal region after rTMS stimulation but also in the apical region at the far end of the stimulus. The study showed that rTMS can affect peripheral close and relatively distant cortical function, and that the area affected by this change is related to the functional state of the brain. The P300 is associated with the cognitive process of conflict resolution. Low-frequency rTMS stimulates the left dorsolateral prefrontal lobe to reduce the amplitude of the P300, indicating that it affects conflict resolution efficiency during task execution and reduces the frontal lobe’s recruitment of cognitive resources [42]. During rTMS treatment, P300 changed from overactivation of the frontal lobe to parietal lobe processing, and the incubation period was shortened. This indicates improved cognitive function, gradually normalized cognitive processing modes, and increased information processing speed. Unfortunately, after treatment, the effects were difficult to maintain.

### 4.4 Limitation

This study had some limitations. The sample size of this study was small, and more participants will be recruited for related experiments in the future. Second, cognitive function includes a variety of subcomponents, including attention, memory, decision-making, logical reasoning, etc. We will further explore the characteristic brain regions of cognitive function through high spatial resolution neuroimaging techniques such as magnetic resonance, and use rTMS for neuromodulation therapy to further confirm the optimal treatment parameters and treatment duration.

### 4.5 Summary

Brain stimulation can causally target local brain regions and affect the activity of the remotely associated regions. Combining rTMS with ERPs allows for a new causal approach to investigate the interactions between extended networks of brain regions during cognition. ERPs induced by the P3-GO and NOGO paradigms appear to be sensitive tools. ERPs can be effective tools for the early monitoring of neurocognitive dysfunction [43]. The right DLPFC is a key factor in the regulation of cognition and emotion, and inhibition of the right DLPFC by low-frequency rTMS stimulation can improve the cognitive processing process of young patients with brain trauma, as well as overall cognitive function, sustained attention ability, and inhibition ability. The effect in the parietal area is obvious, but it has little impact on the face and pattern perception stage, which has positive significance for the evaluation and treatment of brain trauma patients with hidden cognitive dysfunction in the clinic.

## 5 Conclusion

This is a common case of adolescent brain trauma. However, in the clinic, cognitive damage in such patients is often overlooked. This case demonstrates a new method for rehabilitating cognitive impairment in clinical brain trauma. ERPs can be used as biomarkers of cognitive recovery. rTMS has a positive effect on noninvasive regulation and neuroplasticity after brain injury, and the combination of rTMS and ERPs is the direction of attention in future clinical work, improving patients’ cognitive function, depressed emotions, improving patients’ self-care ability, and reducing nursing burden.
